# Nutritional Status as a Risk Factor for Appendiceal Perforation in Pediatric Acute Appendicitis: Systematic Review

**DOI:** 10.3390/children13030326

**Published:** 2026-02-26

**Authors:** Ciprian-Ioan Borca, Cristiana-Smaranda Ivan, Corneluta Fira-Mladinescu, Roxana Margan, Madalin-Marius Margan, Alexandru Cristian Cindrea, Claudia-Raluca Balasa-Virzob, Brigitha Vlaicu, Vlad-Laurentiu David

**Affiliations:** 1 Doctoral School, Victor Babes University of Medicine and Pharmacy Timisoara, E. Murgu Square, No. 2, 300041 Timisoara, Romania; borca.ciprian@umft.ro (C.-I.B.); smaranda.ivan@umft.ro (C.-S.I.); alexandru.cindrea@umft.ro (A.C.C.); 2 Department of Pediatric Surgery and Orthopedics, “Louis Turcanu” Emergency Children’s Hospital, 300011 Timisoara, Romania; david.vlad@umft.ro; 3 Discipline of Hygiene, Department of Microbiology, Victor Babes University of Medicine and Pharmacy, 300041 Timisoara, Romania; roxana.margan@umft.ro (R.M.); vlaicu@umft.ro (B.V.); 4 Center for Studies in Preventive Medicine, Victor Babes University of Medicine and Pharmacy, 300041 Timisoara, Romania; 5 Discipline of Public Health, Department of Functional Sciences, Victor Babes University of Medicine and Pharmacy, 300041 Timisoara, Romania; 6 Centre for Translational Research and Systems Medicine, Faculty of Medicine, Victor Babes University of Medicine and Pharmacy, 300041 Timisoara, Romania; 7 Emergency Department, Emergency Clinical Municipal Hospital, 300254 Timisoara, Romania; 8 Department of Clinic Nursing, Victor Babes University of Medicine and Pharmacy, 300041 Timisoara, Romania; virzob.claudia@umft.ro; 9 Department of Pediatric Surgery and Orthopedics, Victor Babes University of Medicine and Pharmacy, 300041 Timisoara, Romania

**Keywords:** pediatric appendicitis, appendiceal perforation, complicated appendicitis, nutritional status, body mass index, underweight, obesity, serum albumin

## Abstract

**Highlights:**

**What are the main findings?**

- Body Mass Index (BMI) percentiles do not appear to serve as consistent independent predictors of appendiceal perforation or complicated appendicitis in pediatric populations. While obesity is frequently associated with increased operative complexity and healthcare utilization, adjusted analyses have not consistently demonstrated it as an independent predictor of transmural necrosis or perforation. In contrast, underweight status emerges as a more consistent marker of clinical vulnerability, correlating with prolonged hospitalization and increased postoperative morbidity.
- More consistent associations were observed with biochemical nutrition–inflammation indices, such as the CRP/albumin ratio and the neutrophil percentage-to-albumin ratio (NPAR). These markers offer improved discriminatory capacity, as they reflect dynamic physiological processes including capillary leak and hepatic reprioritization during advanced appendiceal inflammation.

**What are the implications of the main findings?**

- These findings suggest that risk stratification in pediatric appendicitis benefits from shifting emphasis from static anthropometric measures toward assessment of physiological reserve and inflammatory status. Integrating albumin-based or composite biochemical markers into existing diagnostic tools, such as the Pediatric Appendicitis Score, may enhance early identification of higher-risk patients.
- The age-dependent variability observed in biomarker performance further suggests that risk-stratification models could be tailored to specific pediatric developmental groups. Additionally, the review highlights that while nutritional vulnerability may influence outcomes, standardized protocol-driven care pathways appear capable of mitigating disparities associated with reduced physiological reserve.
- Overall, these findings support the development of integrated predictive frameworks combining biochemical indicators, clinical severity measures, and temporal factors to improve individualized management in pediatric appendicitis.

**Abstract:**

Background: The association between nutritional status and perforation or complicated appendicitis in children remains uncertain. Objective: To review evidence on anthropometric and biochemical nutritional indicators in relation to perforation and complicated appendicitis in pediatric acute appendicitis. Methods: PubMed, Scopus, and Web of Science were searched for peer-reviewed English-language studies published from 1 January 2010 to 1 January 2026, with supplementary citation searching and Google Scholar screening. Eligible studies included participants aged 0–18 years and reported BMI-based measures and/or biochemical nutritional markers (e.g., albumin, prealbumin, or derived inflammation–nutrition indices) stratified by perforation or complicated appendicitis. Risk of bias was assessed using ROBINS-E. Results: Fourteen observational studies were included. Associations between obesity and perforation or complicated appendicitis were inconsistent, and large registry-based analyses did not identify obesity as an independent predictor after adjustment. Underweight status was more consistently associated with complicated disease and adverse clinical course. Biochemical markers and inflammation–nutrition indices showed more consistent associations with perforated or complicated appendicitis than BMI categories, with several studies reporting moderate-to-high discrimination for severe disease. Conclusions: BMI-based classifications alone did not reliably predict perforation or complicated appendicitis. Albumin- and prealbumin-based indices were more consistently associated with disease severity, but the observational evidence does not establish causality and may reflect inflammatory severity at presentation. Prospective studies with standardized definitions and marker assessment are needed to evaluate incremental prognostic value beyond symptom duration and clinical severity scores.

## 1. Introduction

Acute appendicitis is one of the most frequent pediatric surgical emergencies globally, representing a significant source of morbidity and healthcare costs [1,2,3,4,5]. The traditional perspective held that appendiceal inflammation followed a predetermined path toward rupture, with surgery seen as the only means of prevention [6,7]. However, this view has been updated by modern data characterizing appendicitis as a spectrum of situations [8,9]. Complicated appendicitis is a hierarchical category that includes perforation but is not limited to it; it generally encompasses perforation, periappendiceal abscess, phlegmon, and generalized peritonitis [10,11]. Perforation therefore represents a specific pathological event within the complicated spectrum and may be considered a subset of complicated appendicitis.

The clinical course of pediatric appendicitis varies at the time of presentation. In uncomplicated cases, inflammation remains localized and follows a relatively mild trajectory, allowing for nonoperative management with antibiotics in selected patients [12,13]. In contrast, complicated appendicitis is characterized by tissue necrosis and diffuse inflammatory spread, a progression associated with a threefold increase in complications and significantly prolonged recovery [14,15,16]. Perforated appendicitis refers specifically to transmural necrosis of the appendiceal wall with macroscopic or radiologic evidence of wall defect and communication between the lumen and the peritoneal cavity [17].

In Europe, appendicitis remains one of the most common pediatric surgical emergencies, with reported incidence rates ranging from 105 to 151 cases per 100,000 children per year [18]. Despite its frequency, a central question in pediatric care remains unresolved: why does the risk of advanced disease peak at younger ages, when the overall incidence of appendicitis is lowest? One contributing factor may be anatomical. In early childhood, the greater omentum—an important peritoneal defense mechanism—often lacks sufficient length and adipose content to effectively contain localized inflammation [19]. This developmental limitation reduces the capacity for compartmentalization, permitting a focal appendiceal infection to progress more rapidly to generalized peritonitis.

Such vulnerability suggests that, in younger children, factors beyond anatomy and imaging may play a decisive role in disease progression. Nutritional status may represent one such factor. Obesity has been associated with a pro-inflammatory state, while malnutrition may impair tissue integrity and immune response, potentially influencing the resilience of the appendiceal wall [20,21]. Despite advances in ultrasonography and magnetic resonance imaging, accurately predicting progression to perforation remains challenging [22,23].

Globally, pediatric nutritional disorder prevalence differs by region [24,25]. In many developing countries, protein–energy malnutrition and micronutrient deficiencies impair immune competence and tissue repair, prolonging postoperative convalescence [25]. Conversely, childhood obesity has risen substantially worldwide, including in Europe, resulting in a need for larger surgical sections and prolonged surgical time [26,27,28].

Beyond these clinical observations, the immunometabolic state of the pediatric patient influences the trajectory and degree of the inflammatory response. Previous studies evaluating inflammatory biomarkers such as interleukin-6 (IL-6), C-reactive protein (CRP), and white blood cell (WBC) levels show predictive potential of complicated appendicitis and display associations between elevated inflammatory markers and perforation risk [29,30,31,32]. In obese children, adipose tissue secretes pro-inflammatory cytokines, including IL-6 and tumor necrosis factor-alpha (TNF-α), which may contribute to accelerated tissue injury within the confined space of the appendiceal lumen [33,34,35]. Conversely, micronutrient deficiencies and protein–energy malnutrition can impair collagen synthesis and compromise mucosal barrier integrity, potentially lowering the threshold for bacterial translocation and subsequent perforation [36,37]. Together, these mechanisms suggest that nutritional status can meaningfully impact disease severity alongside traditional clinical factors.

Current risk-stratification tools, such as the Pediatric Appendicitis Score (PAS) or the Alvarado score, rely heavily on clinical signs and leukocyte counts, which often overlap between uncomplicated and complicated phenotypes [22,38,39]. While these tools are effective for diagnosis, they lack the sensitivity to reliably predict perforation in its earliest stages. Furthermore, although preoperative C-reactive protein (CRP) levels provide some prognostic value, they do not account for the host’s underlying physiological resilience [22,29]. Integrating nutritional markers—such as Body Mass Index (BMI) percentiles, serum albumin, or specific vitamin levels—into these predictive models could provide a more personalized approach to surgical triage, yet these variables are rarely standardized in existing pediatric protocols.

This systematic review seeks to offer a view of the current state of perforated appendicitis in children. Growing evidence suggests that nutritional status and immunometabolic factors may influence the risk of perforation [40,41,42,43,44,45], yet current risk-stratification tools rely primarily on clinical signs and basic laboratory tests and often fail to predict perforation reliably at presentation. By synthesizing the fragmented literature on nutritional markers in pediatric appendicitis, this review aims to provide an evidence-based view meant to inform more personalized risk assessment and early triage, making it relevant in the context of rising childhood obesity and persistent malnutrition worldwide.

## 2. Materials and Methods

A systematic literature search was conducted in PubMed, Scopus and Web of Science using query search, while Google Scholar search engine was used as a supplementary search with natural language (as Google Scholar does not support controlled vocabularies, field-specific searching, complex Boolean nesting, or reproducible indexing [46]), scoping for papers which might have been missed due to intrinsic search engine limitations (retrieval, indexing and terminology limitations). The literature search used structured, database-specific Boolean queries combining terms for acute appendicitis, appendiceal perforation, pediatric populations, and nutritional status. This was performed to identify relevant studies published in English between 1 January 2010 and 1 January 2026. Only published, peer-reviewed studies were considered; unpublished studies and gray literature were not retrieved.

All records identified through database searching and other sources were screened independently by at least two reviewers (C.-I.B. and C.B.), and if differences regarding inclusion/exclusion were raised, conciliation was performed by a third independent reviewer (M.-M.M.) in discussion with the first two and in accordance with the eligibility criteria as described below. Titles and abstracts were first assessed for potential eligibility, followed by full-text review of selected articles.

Studies were selected according to predefined eligibility criteria based on the Population, Exposure, Comparator, Outcome, and Study design (PECOS) framework [47]. Eligible studies included human children and adolescents aged 0–18 years, or studies using a pediatric definition from which results for participants younger than 18 years could be extracted separately. Participants were required to have a diagnosis of acute appendicitis with a clearly stated diagnostic basis, and outcomes had to be stratified by appendicitis outcome. Studies were required to report at least one nutritional status indicator measured or recorded at or before presentation, including anthropometric measures (e.g., BMI or BMI categories) and/or nutrition-related biochemical markers such as serum albumin or total protein.

Adult-only studies, mixed adult–pediatric cohorts without extractable pediatric data, studies not reporting perforation outcomes, studies lacking measurable nutritional status indicators, and studies with unclear case or perforation definitions were excluded. Case reports, non-comparative case series, narrative reviews, editorials, commentaries, conference abstracts without sufficient data, animal studies, and laboratory studies were also excluded.

The primary outcome was appendiceal perforation in pediatric acute appendicitis, defined as perforated versus non-perforated disease according to each study’s reported criteria. Study findings were synthesized systematically, and when sufficient comparability existed across studies in terms of nutritional status measures and perforation definitions, structured tables were used to summarize key study characteristics and results, including exposure definitions, outcome definitions, and reported effect estimates.

This systematic review was conducted and reported in accordance with the Preferred Reporting Items for Systematic Reviews and Meta-Analyses (PRISMA) 2020 statement (Centre for Reviews and Dissemination, University of York, York, UK) [48] and followed a protocol prospectively registered in PROSPERO (CRD420261292734).

Data extraction was performed independently by two reviewers (I.C.-S. and R.M.) using a systematic data extraction process. Extracted data included study characteristics (study design, setting, country, and sample size), participant characteristics, definitions of appendiceal perforation, nutritional status indicators and their categorization, comparator definitions, and reported measures of association between nutritional status and perforation.

Risk of bias was assessed independently by two reviewers (I.C.-S. and C.-R.B.-V.). For exposure-based observational studies, the ROBINS-E tool (University of Bristol, Bristol, UK) was used. Disagreements were resolved by consensus. Risk-of-bias judgments were visualized using the robvis tool (Supplementary Figures S1 and S2). Bias due to selective reporting or missing results was considered within the ROBINS-E domain “bias in selection of the reported result” and was not assessed using a separate reporting-bias tool [49].

Meta-analysis was not performed due to substantial heterogeneity in exposure definitions, outcome measures, and study designs across the included studies.

## 3. Results

### 3.1. Overview of the Included Studies

**Figure 1:**
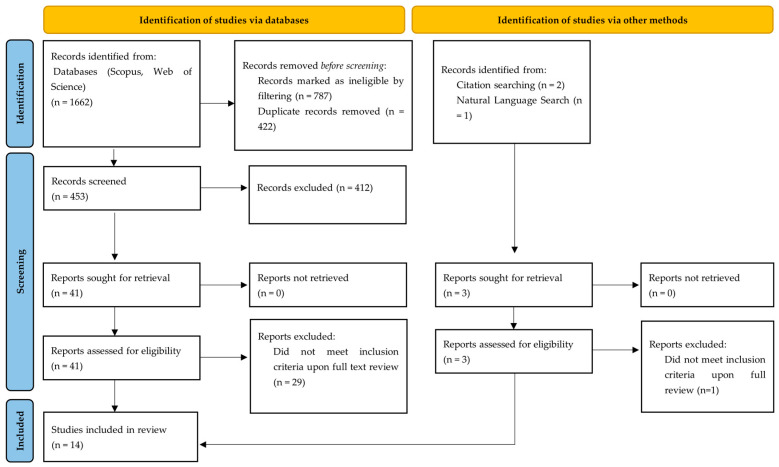
PRISMA 2020 flow diagram, which includes searches of databases and other sources.

Following the search methodology, the systematic literature search across Scopus, Web of Science and PubMed identified 1662 records. After application of database filters, 41 records were excluded prior to screening due to non-English language, and 746 records were excluded based on publication type (e.g., reviews, editorials, conference abstracts, animal or laboratory studies). After removal of 422 duplicate records, 453 unique articles remained and were subjected to title and abstract screening. Of these, 41 articles underwent full-text review for eligibility, resulting in 12 studies from database searching. As additional sources, backward citation searching identified two additional reports, and supplementary natural-language searching using Google Scholar identified one additional report. Following full-text assessment, one report was excluded for not meeting the predefined inclusion criteria. In total, 14 studies were included in the final synthesis, with full breakdown of the selection process available in the PRISMA flow diagram (Figure 1).

This systematic review included 14 observational studies evaluating the association between nutritional status and appendiceal perforation or complicated appendicitis in pediatric patients. Sample sizes ranged from 74 to 23,153 children, encompassing both single-center cohorts and large national database studies. All included studies involved pediatric populations (≤18 years), with mixed-sex cohorts.

Most studies employed retrospective cohort designs (n = 11), while the remaining studies were prospective or cross-sectional (n = 3). The studies were conducted across multiple regions, including North America, Europe, Asia, and the Middle East, reflecting a broad geographic distribution.

Disease severity was defined as perforated or complicated appendicitis using intraoperative findings, pathological confirmation, or standardized registry definitions, typically including perforation, gangrene, abscess formation, or generalized peritonitis. Most studies stratified outcomes as complicated versus uncomplicated appendicitis.

Nutritional status was assessed using anthropometric and biochemical indicators. BMI-based measures were reported in nine studies, with patients categorized as underweight, normal weight, overweight, or obese using age-adjusted percentiles. Five studies evaluated biochemical nutritional markers, including serum albumin, prealbumin, and composite indices such as the C-reactive protein–to–albumin ratio, neutrophil percentage–to–albumin ratio, and prognostic nutritional index.

Overall, several studies reported higher rates of perforation or complicated appendicitis among underweight children, whereas findings related to obesity were inconsistent across cohorts. Studies incorporating biochemical markers consistently demonstrated associations between lower nutritional indices or higher inflammation–nutrition ratios and increased disease severity. The characteristics and main findings of the included studies are summarized in Table 1.

### 3.2. Anthropometric Indicators of Nutritional Status

#### 3.2.1. Obesity and Perforation

Two early retrospective cohort studies specifically examined the relationship between obesity and appendiceal perforation with differing results. Blanco et al. (n = 319) reported that obese children had significantly higher odds of presenting with perforated appendicitis compared with non-obese peers, with obesity remaining an independent predictor after multivariable adjustment (adjusted OR ≈ 2.0, *p* < 0.05) [41]. In contrast, Ramos et al. (n = 170) observed higher unadjusted perforation rates among obese children; however, this association was attenuated and no longer statistically significant after adjustment, suggesting that obesity alone may not independently drive perforation risk [42].

#### 3.2.2. Underweight Status and Disease Severity

In contrast to obesity, underweight status was more consistently associated with overall disease severity rather than perforation alone. Aslan et al. (n = 96) found no statistically significant association between BMI category and appendiceal perforation, although children with high BMI experienced significantly higher postoperative complication rates [51]. Supporting this observation in a larger cohort, Hebballi et al. (n = 23,153) demonstrated that underweight children had significantly increased odds of complicated appendicitis compared with normal-weight peers (OR = 1.66; 95% CI: 1.06–2.59) [45].

Further evidence comes from Timmerman et al. (n = 457), who found no direct association between BMI category and perforation; however, underweight children experienced higher complication rates and longer hospital stays, suggesting increased clinical vulnerability even in the absence of higher perforation frequency [43].

#### 3.2.3. Registry-Based Analyses of Complicated Appendicitis

Two large database studies evaluated BMI and composite definitions of complicated appendicitis. In the ACS NSQIP–Pediatric cohort, Michailidou et al. (n = 2812) reported that obesity was not independently associated with complicated appendicitis after multivariable adjustment [52]. Similarly, Hebballi et al. found no independent association between obesity and complicated appendicitis, while overweight status was paradoxically associated with reduced odds of severe disease (OR = 0.72; 95% CI: 0.54–0.95) [45].

The results of these studies are summarized in Table 2.

### 3.3. Malnutrition-Based Nutritional Assessments

Evidence from a limited number of studies suggests that malnutrition-related nutritional indicators, particularly biochemical markers, may be associated with the severity of pediatric appendicitis.

In a cross-sectional study from Bangladesh, Chowdhury et al. (n = 155) evaluated a panel of anthropometric and biochemical nutritional indicators in children with acute appendicitis. While anthropometric measures—including BMI-for-age, mid-upper arm circumference, and skinfold thickness—did not differ significantly between uncomplicated and complicated appendicitis, serum albumin levels were significantly lower in children with complicated appendicitis (*p* < 0.001). Moderate-to-severe hypoalbuminemia was markedly more prevalent among complicated cases, supporting an association between impaired nutritional reserve and advanced disease severity.

Complementing these findings, Banlı-Cesur et al. [44] (n = 74) assessed nutritional status using the Gomez malnutrition classification alongside BMI-derived cut-offs. Although malnutrition and low BMI were associated with worse clinical outcomes—including longer hospital stay and higher wound infection rates—no independent association with perforated or complicated appendicitis was demonstrated. Disease severity was inferred indirectly through postoperative morbidity rather than operative confirmation of perforation, limiting direct comparability with other cohorts.

Together, these studies suggest that biochemical manifestations of malnutrition, particularly hypoalbuminemia, may be more closely associated with complicated appendicitis than anthropometric malnutrition alone, while formal malnutrition classifications appear more strongly linked to postoperative morbidity than to perforation risk itself. The characteristics and key findings of these studies are summarized in Table 3.

### 3.4. Biochemical Nutritional and Inflammation-Based Markers

Evidence increasingly supports nutrition–inflammation biomarkers—particularly albumin- and prealbumin-based indices—as useful tools for identifying children at higher risk of complicated appendicitis (including gangrene/perforation) and perforated appendicitis specifically.

Two studies evaluating albumin-based inflammation ratios reported strong and consistent associations with complicated diseases. In a retrospective cohort, Hou et al. (2022) [54] (n = 296) found the CRP/albumin (CRP/ALB) ratio to be significantly higher in complicated versus simple appendicitis (*p* < 0.05), and multivariable logistic regression confirmed the CRP/ALB ratio as an independent risk factor (OR = 6.92, 95% CI 3.21–14.94), while albumin itself was protective (OR = 0.63, 95% CI 0.53–0.74). Diagnostic performance was high (AUC = 0.883), and a CRP/ALB cut-off ≥ 1.39 yielded 86.6% sensitivity and 84.6% specificity; children above this cut-off had markedly higher odds of complicated appendicitis (OR = 31.26, 95% CI 16.45–59.42) [54]. Similarly, Liu et al. (2025) [56] (n = 814) demonstrated that the neutrophil percentage-to-albumin ratio (NPAR) was associated with complicated appendicitis after adjustment for key confounders (fully adjusted OR per unit increase = 1.27, 95% CI 1.15–1.39, *p* < 0.001). Importantly, the relationship was non-linear, with an identified threshold of 19.53: below this level, each unit increase in NPAR was strongly associated with increased risk (OR = 1.46, 95% CI 1.27–1.68, *p* < 0.001). Discrimination was good (AUC = 0.811, 95% CI 0.777–0.844) [56].

A related line of evidence focuses on prealbumin, a marker reflecting short-term nutritional status and acute inflammation. In a cross-sectional surgical cohort, Long et al. (2024) [55] (n = 313) showed that the CRP/prealbumin ratio (CPA) differed markedly between perforated and non-perforated appendicitis (median 6.63 vs. 0.70, *p* < 0.001) and remained independently associated with perforation in multivariable analysis (OR = 5.55, 95% CI 1.73–17.83, *p* = 0.004). Diagnostic performance was notably age-dependent, reaching AUC 0.816 in children aged 4–9 years and 0.919 in those aged 9–16 years using reported cut-offs [55].

Extending beyond single markers, a composite “nutrition + immune + inflammation” approach is supported by Tusat et al. (2025) [57] (n = 253), who found that children with complicated appendicitis had significantly lower PNI (*p* = 0.027) and HALP score (*p* = 0.007), alongside higher inflammatory ratios (NLR, *p* = 0.003; PLR, *p* = 0.008). While the study primarily reports group comparisons rather than adjusted odds ratios, the directionality aligns with the broader pattern that lower nutritional reserve plus higher inflammatory burden accompanies complicated disease [57].

All findings described here are summarized in Table 4.

## 4. Discussion

This systematic review aimed to synthesize the current evidence on the role of anthropometric and nutritional indicators in predicting disease severity in pediatric appendicitis. Specifically, we sought to address the following question: to what extent do BMI-based measures and nutritional markers predict perforation, complicated appendicitis, postoperative morbidity, and clinical course in children with acute appendicitis?

### 4.1. Potential Influence of Nutritional Status on Appendiceal Tissue Integrity

Nutritional status may influence the balance between inflammatory injury and tissue resistance by modulating extracellular matrix (ECM) turnover, immune competence, and microvascular function, all of which can affect progression from acute inflammation to transmural necrosis and perforation [58]. Undernutrition and protein deficiency are known to impair collagen synthesis, reduce fibroblast proliferation, and weaken extracellular matrix organization, resulting in decreased tensile strength of tissues [37]. Experimental data demonstrate that protein–energy malnutrition compromises intestinal barrier function and delays mucosal repair, increasing susceptibility to transmural injury under inflammatory stress [59]. While duration of inflammation, bacterial proliferation, and intraluminal pressure remain primary determinants of perforation, these host-related mechanisms increase tissue vulnerability and therefore contribute to disease progression in susceptible pediatric patients [60].

Conversely, overweight and obesity may influence inflammatory responses through distinct immunometabolic pathways. Obesity is characterized by adipose tissue expansion requiring extracellular matrix (ECM) remodeling, and maladaptive or insufficient “healthy” expansion has been associated with adipocyte necrosis, hypoxia, chronic low-grade inflammation, fibrosis, and insulin resistance [61,62]. Mechanistically, if systemic inflammatory tone is higher, an acute intra-appendiceal inflammatory process could plausibly reach a more destructive threshold (e.g., faster progression to transmural necrosis), although this specific progression has not been directly demonstrated in pediatric appendicitis and should be regarded as hypothesis-generating.

However, when these biologically plausible pathways are interpreted considering the findings synthesized in the present review, the available clinical evidence appears to lean more consistently toward markers of poor nutritional status as being associated with a higher risk of appendiceal perforation than overweight or obesity.

### 4.2. Prognostic Value of BMI and Anthropometric Measures

Across the available literature, obesity does not emerge as a consistent independent predictor of perforation or complicated appendicitis. While an early cohort suggested a possible association [41], this finding was not consistently replicated in later cohorts and registry-based analyses after multivariable adjustment [42,45,52]. Broader syntheses of the literature further support this interpretation. Systematic reviews by Zavras et al. and Rivero-Moreno et al. concluded that obesity is variably associated with perioperative outcomes such as operative time, length of stay, and surgical-site infection, but does not reliably predict perforation or severe disease [28,63]. Similarly, Papillon et al. reported no independent association between obesity and complicated appendicitis once clinical confounders were considered [64]. Overall, across adjusted cohort analyses and registry-based studies, the evidence consistently fails to demonstrate obesity as an independent predictor of perforation.

This inconsistency is biologically plausible. BMI is a static, population-level measure that does not capture acute inflammatory burden, immune activation, or metabolic reserve—factors central to progression from uncomplicated appendicitis to gangrene or perforation. In addition, obesity-related inflammation is typically chronic and low grade, differing fundamentally from the acute infectious and ischemic processes that characterize advanced appendicitis [65,66,67,68]. Clinically, obesity may complicate physical examination, increase reliance on imaging, and influence perioperative management decisions, thereby affecting healthcare utilization rather than underlying disease biology, as suggested by observational studies and pathway-based analyses.

### 4.3. Underweight Status as a Marker of Physiological Vulnerability

In contrast to obesity, underweight status appears to reflect host vulnerability rather than an increased propensity for perforation. Large cohort studies have shown that underweight children experience more severe clinical courses, including longer hospital stays and higher postoperative complication rates, even when perforation rates are not significantly increased [38,42].

This pattern aligns with broader conceptual models of pediatric appendicitis severity, which emphasize that clinical outcomes are shaped by the interaction between inflammatory burden and physiological reserve rather than anatomical findings alone. For example, the Pediatric Appendicitis Severity Assessment (PASAP) model described by Howell et al. [69] conceptualizes disease progression as a dynamic process in which a high inflammatory load can overwhelm host reserve, leading to perforation irrespective of gross anatomic appearance on imaging. In validated cohorts, PAS predicted complex appendicitis more accurately than traditional scoring systems such as the Alvarado score (AUC 0.85), prioritizing laboratory trends and physiologic responses over static patient characteristics [8,70].

This interactionist framework is further supported by nomograms and machine-learning models that integrate symptom duration, persistent fever, and weight-for-age to forecast disease severity, with decision curve analyses favoring early intervention across intermediate risk thresholds (14–88%) [71,72,73]. Collectively, these findings reinforce a model in which vulnerability arises from the balance between inflammatory stress and physiological reserve, consistent with the heightened risk observed in underweight children and the predominantly indirect influence of obesity discussed earlier.

This pattern suggests that a child’s physiological reserve—their ability to withstand the systemic stress of anesthesia and the catabolic demands of infection—is a more critical determinant of outcomes than anatomical findings alone. Children with limited metabolic and immunological reserves are simply less resilient to the inflammatory storm, a conceptual model that shifts our focus from “body size” to “biological capacity.”

### 4.4. Added Value of Biochemical Nutritional Markers

The limitations of anthropometric measures are most evident when compared to the predictive power of biochemical nutritional indicators. Research consistently demonstrates that while BMI-based classifications fail to independently predict disease severity, biochemical abnormalities—most notably hypoalbuminemia—provide a more sensitive signal of risk.

Studies by Chowdhury et al. and Banlı-Cesur et al. demonstrated that anthropometric measures were not independently associated with complicated appendicitis, whereas biochemical abnormalities—particularly hypoalbuminemia—were more consistently linked to disease severity and adverse outcomes [44,53].

This biochemical superiority is rooted in two critical mechanisms: hepatic reprioritization and capillary leak [67,70]. During advanced infection, the liver undergoes a metabolic shift, redirecting protein synthesis away from constitutive proteins like albumin and toward acute-phase reactants necessary for immune defense [74,75,76]. Consequently, a drop in serum albumin acts as a sign for the intensity of this systemic inflammatory shift [74,75,76,77,78]. Simultaneously, severe appendicitis triggers increased vascular permeability, allowing albumin to escape the intravascular space [79,80]. This process, known as capillary leak, directly correlates with the transition from localized inflammation to complicated, systemic diseases, a shift that remains invisible to traditional measurements of height and weight [81,82].

These findings suggest that biochemical markers capture dynamic nutritional–inflammatory stress, integrating hepatic reprioritization of protein synthesis, cytokine-driven catabolism, and capillary leak—processes that are central to advanced appendiceal disease but invisible to static anthropometric assessments.

### 4.5. Interaction with Health-System Factors and Standardized Care

Importantly, the effects of anthropometric and nutritional factors on clinical outcomes appear modifiable through system-level interventions. Evidence from standardized care pathways and quality-improvement initiatives suggests that protocol-driven care can reduce outcome disparities across BMI and nutritional categories by minimizing variability in antibiotic timing, operative management, postoperative care and resources [83,84,85,86]. Similar benefits of standardized protocols have been observed across pediatric surgical populations, where structured care pathways mitigate risk related to physiological vulnerability rather than baseline body size alone [87,88].

In pediatric appendicitis, Yousef et al. demonstrated significant reductions in postoperative morbidity following implementation of standardized pathways for perforated disease [87]. Complementary studies reported that omission of low-value interventions did not increase complication rates while reducing length of stay and resource utilization [86,88]. Together with broader pathway-based and ERAS-aligned analyses, these findings suggest that timely, standardized care may show a greater influence on outcomes than anthropometric risk alone, reframing body size as a modifiable rather than deterministic factor.

### 4.6. Clinical Implications and Future Directions

Taken together, the results of this review suggest that anthropometric measures alone are insufficient for reliable risk stratification in pediatric appendicitis. Biochemical nutritional and inflammation-based markers offer a biologically plausible and clinically actionable complement to traditional assessment, potentially enabling earlier identification of high-risk patients and more tailored perioperative management.

Future prospective studies should aim to integrate biochemical markers, anthropometric measures, clinical severity scores, and social determinants of health within unified predictive models. Such approaches may improve diagnostic accuracy, guide resource allocation, and ultimately reduce morbidity in children with complicated appendicitis.

Beyond disease severity itself, nutritional status influences perioperative management. In obese pediatric patients, appendectomy is generally more demanding, technically speaking, requiring longer operative times and greater surgical exposure. In addition, undernutrition and hypoalbuminemia impair postoperative wound healing due to compromised tissue repair and immune function. Although these factors do not establish a direct causal relationship between nutritional status and appendiceal perforation, they underscore the broader clinical relevance of nutritional assessment in pediatric surgical care.

### 4.7. Limitations

Our study presents the following limitations. Variability in study design, exposure definitions, outcome measures, and biomarker cut-offs limits direct comparison across cohorts contribute to clinical heterogeneity. In addition, most available studies are retrospective, increasing susceptibility to selection bias and residual confounding.

Importantly, the current literature lacks large cohort, prospective studies, specifically designed to determine whether nutritional status—particularly obesity or malnutrition—independently influences the risk of appendiceal perforation. Such studies would ideally control for major confounding variables, such as age, duration of symptoms, timing of surgical intervention, intraluminal bacterial burden, and appendiceal wall pressure. In the absence of this level of methodological rigor, observed associations between nutritional status and perforation should be interpreted as exploratory rather than causal. Worth keeping in mind is that the timing of biomarker measurement was inconsistently reported across studies and may substantially influence observed associations, particularly in a rapidly evolving inflammatory condition such as acute appendicitis.

Potential publication bias must also be considered, as studies reporting significant associations between nutritional markers and disease severity may be more likely to be published than null findings.

Multicenter prospective investigations that integrate clinical severity, temporal factors, microbiological variables, and nutritional–inflammatory biomarkers are required to better elucidate potential mechanistic pathways and improve risk stratification.

## 5. Conclusions

Across studies using anthropometric measures, obesity was not a consistent independent predictor of perforation or complicated appendicitis after adjustment for confounders, with findings varying by cohort and outcome definition.

Underweight status was more consistently associated with complicated appendicitis or a more adverse clinical course. Biochemical and nutrition–inflammation indices incorporating albumin or prealbumin (e.g., CRP/albumin ratio, neutrophil percentage-to-albumin ratio, CRP/prealbumin ratio, PNI, HALP) showed more consistent associations with complicated or perforated appendicitis than BMI-based classifications, and several studies reported useful discrimination for severe disease.

Given the observational design, heterogeneity in definitions, and frequent use of biomarkers measured at presentation, these findings support association rather than causation and do not establish whether low nutritional indices precede severe disease or reflect inflammatory severity. Future prospective studies should evaluate standardized nutritional and inflammatory markers alongside symptom duration and validated clinical scores and assess whether adding biochemical nutritional indices improves early risk stratification beyond existing approaches. Importantly, standardized care pathways can mitigate outcome disparities across nutritional categories, highlighting the modifiable nature of these risks.

Together, these findings support a shift from static anthropometric assessment toward integrated models incorporating biochemical markers, clinical severity, and health-system factors to improve risk stratification and outcomes in pediatric appendicitis.

## Figures and Tables

**Table 1:** Overview of the studies included.

Author (Year)	Study Design	Country	Number of Patients	Definition of Perforation/Complicated Appendicitis	Nutritional Exposure(s) Assessed	Comparator Group	Key Conclusions	Reference Number
Garey (2011)	Analysis of pooled prospective trials	USA	220	Intraoperative finding of appendiceal hole or fecalith in abdomen	BMI percentile (obesity ≥ 95th percentile)	Obese vs. non-obese	Obese children with perforated appendicitis had longer operative times, longer length of stay, and higher abscess rates.	[40]
Sulowski (2011)	POC	Canada	263	Perforation identified at surgery or imaging	BMI-for-age percentile (≥85th percentile)	Obese vs. normal weight	Obesity did not increase perforation rates or complications but was associated with higher CT utilization.	[50]
Aslan (2012)	ROC	Turkey	96	Acute vs. perforated appendicitis based on operative findings	BMI percentiles (low, normal, high)	Low vs. normal vs. high BMI	High BMI was associated with increased postoperative complications; perforation rates differed across BMI groups.	[51]
Blanco (2012)	ROC	USA	319	Operative diagnosis of perforated vs. non-perforated appendicitis	BMI percentile (obesity ≥ 95th percentile)	Obese vs. non-obese	Children with obesity were more likely to present with perforated appendicitis.	[41]
Ramos (2012)	ROC	Puerto Rico	171	Surgical diagnosis of perforation	BMI-for-age percentile	Obese vs. non-obese	Obesity showed a tendency toward higher perforation risk, though not independently significant after adjustment.	[42]
Michailidou (2015)	Database study (ACS NSQIP-P)	USA	2812	ICD-9 coding of complicated vs. uncomplicated appendicitis	BMI percentile (obesity ≥ 95th percentile)	Obese vs. non-obese	Obesity was not an independent predictor of postoperative complications but was associated with longer operative time.	[52]
Timmerman (2016)	ROC	Netherlands	457	Intraoperative identification of perforation	BMI percentiles (underweight, normal, overweight, obese)	Underweight/overweight/obese vs. normal	Underweight children had higher complication rates and longer hospital stays; BMI did not significantly affect perforation rates.	[43]
Banlı-Cesur (2022)	ROC	Turkey	74	Operative diagnosis of appendicitis with postoperative outcomes	BMI and Gomez malnutrition classification	Malnourished vs. well-nourished	Low BMI and malnutrition were associated with increased postoperative morbidity and wound infection.	[44]
Chowdhury (2022)	CS	Bangladesh	155	Intraoperative classification into uncomplicated vs. complicated appendicitis	Anthropometry, serum albumin, hemoglobin, TLC	Uncomplicated vs. complicated appendicitis	Malnutrition was more prevalent in complicated appendicitis; low serum albumin was significantly associated with complications.	[53]
Hou (2022)	ROC	China	296	Pathological diagnosis of complicated vs. simple appendicitis	Serum albumin, CRP/albumin ratio	High vs. low CRP/albumin ratio	Elevated CRP/albumin ratio strongly predicted complicated appendicitis in children.	[54]
Hebballi (2023)	ROC	USA	23,153	NSQIP definition of complicated appendicitis (perforation, gangrene, or abscess)	BMI percentiles (underweight, normal weight, overweight, obese)	BMI categories compared with normal-weight children	Underweight children had significantly higher odds of complicated appendicitis, whereas overweight status was associated with lower odds; underweight and obesity were both associated with increased postoperative complications.	[45]
Long (2024)	CS	China	313	Intraoperative and pathological confirmation of perforated vs. non-perforated appendicitis	CRP/prealbumin ratio (CPA), albumin, prealbumin	Perforated vs. non-perforated	Higher CPA values were independently associated with perforated appendicitis, with strong age-stratified diagnostic performance.	[55]
Liu (2025)	ROC	China	814	Pathological classification into uncomplicated vs. complicated appendicitis	Neutrophil percentage-to-albumin ratio (NPAR), albumin	High vs. low NPAR	Elevated NPAR showed a non-linear association with complicated appendicitis and demonstrated good discriminatory performance.	[56]
Tusat (2025)	ROC	Türkiye	253	Pathological or intraoperative classification of complicated vs. non-complicated appendicitis	Prognostic Nutritional Index (PNI), HALP score, albumin-based indices	Complicated vs. non-complicated	Lower PNI and HALP scores were significantly associated with complicated appendicitis, supporting nutritional-inflammation markers as predictors of disease severity.	[57]

ACS NSQIP-P—American College of Surgeons National Surgical Quality Improvement Program–Pediatric; BMI—Body Mass Index; CPA—C-reactive protein to prealbumin ratio; CRP—C-reactive protein; CS—Cross-sectional study; CT—Computed tomography; HALP—Hemoglobin, Albumin, Lymphocyte, and Platelet score; ICD-9—International Classification of Diseases, Ninth Revision; NPAR—Neutrophil percentage-to-albumin ratio; PNI—Prognostic Nutritional Index; POC—Prospective observational cohort; ROC—Retrospective observational cohort; TLC—Total leukocyte count.

**Table 2:** Association between anthropometric measures and complicated appendicitis in children. BMI—Body Mass Index; CI—Confidence interval; OR—Odds ratio.

Author (Year)	Anthropometric Measure	Nutritional Categories	Association with Complicated Appendicitis	Adjusted Analysis	Reference
Garey (2011)	BMI percentile	Obese vs. non-obese	Obesity was not associated with an increased risk of appendiceal perforation; adjusted odds ratios were not reported.	Yes	[40]
Sulowski (2011)	BMI percentile	Obese vs. normal weight	No significant association was observed between obesity and perforated appendicitis (adjusted effect estimates not reported).	Yes	[50]
Aslan (2012)	BMI percentile	Low/normal/high	No significant association was observed between obesity and perforated appendicitis	Not clearly adjusted	[51]
Blanco (2012)	BMI percentile	Obese vs. normal weight	Obesity was independently associated with an increased risk of perforated appendicitis (adjusted OR: ≈2.0).	Yes	[41]
Ramos (2012)	BMI percentile	BMI percentile groups	Obesity was associated with higher odds of perforated appendicitis in unadjusted analyses; however, this association was attenuated and no longer statistically significant after multivariable adjustment (adjusted OR not significant).	Yes	[42]
Michailidou (2015)	BMI percentile	Obese vs. non-obese	Obesity was not independently associated with complicated appendicitis after multivariable adjustment (adjusted OR not significant).	Yes	[52]
Timmerman (2016)	BMI percentile	Underweight/normal/overweight/obese	Body mass index category was not associated with appendiceal perforation, although underweight children experienced a more complicated clinical course (adjusted ORs for perforation not significant).	Yes	[43]
Hebballi (2023)	BMI percentile	Underweight/normal/overweight/obese	Underweight status was significantly associated with an increased risk of complicated appendicitis (OR: 1.66; 95% CI: 1.06–2.59), whereas overweight status was associated with a reduced risk (OR: 0.72; 95% CI: 0.54–0.95). Obesity was not independently associated with complicated appendicitis.	Yes	[45]

**Table 3:** Malnutrition-Based Nutritional Indicators and Risk of Complicated Appendicitis in Pediatric Patients. BMI—Body mass index; MUAC—Mid-upper arm circumference; TSFT—Triceps skinfold thickness; TLC—Total lymphocyte count.

Author (Year)	Malnutrition Indicator	Reference Group	Key Association with Complicated Appendicitis	Reference
Banlı-Cesur (2022)	Gomez malnutrition classification; low BMI (cut-off < 16.74)	Well-nourished children (normal Gomez score/BMI ≥ 16.74)	Mild–moderate malnutrition and low BMI were associated with worse clinical outcomes; however, no independent association with perforated/complicated appendicitis was demonstrated. Low BMI was associated with higher wound infection rates and longer hospital stay, but complication status was defined indirectly by prolonged hospitalization rather than operative perforation	[44]
Chowdhury (2022)	Anthropometric indices (BMI-for-age, MUAC, TSFT, weight-for-age, height-for-age) and biochemical markers (serum albumin, hemoglobin, lymphocyte count)	Children with normal nutritional indices	Serum albumin was significantly associated with complicated appendicitis: moderate–severe hypoalbuminemia was more frequent in complicated cases (*p* < 0.001). No significant association was observed between anthropometric indicators (BMI, MUAC, TSFT) and complicated appendicitis	[53]

**Table 4:** Biochemical nutritional and inflammation-based markers associated with complicated appendicitis in children.

Author (Year)	Biochemical Marker/Index	Marker Components	Key Association with Complicated Appendicitis	Reference
Hou (2022)	CRP/Albumin ratio	C-reactive protein, serum albumin	CRP/albumin ratio was significantly higher in complicated appendicitis (*p* < 0.05) and independently associated with disease severity (OR = 6.92; 95% CI 3.21–14.94). A cut-off ≥ 1.39 predicted complicated appendicitis with 86.6% sensitivity and 84.6% specificity (AUC = 0.883). Albumin was protective (OR = 0.63; 95% CI 0.53–0.74).	[54]
Long (2024)	CPA	C-reactive protein, prealbumin	CPA values were significantly higher in perforated appendicitis than in non-perforated cases (median 6.63 vs. 0.70; *p* < 0.001). CPA independently predicted perforation (OR = 5.55; 95% CI 1.73–17.83). Diagnostic performance was age-dependent, with AUCs up to 0.919 in older children.	[55]
Liu (2025)	NPAR	Neutrophil percentage, serum albumin	NPAR showed a significant non-linear association with complicated appendicitis. Below a threshold of 19.53, each unit increase was associated with higher risk (OR = 1.46; 95% CI 1.27–1.68; *p* < 0.001). Overall discrimination was good (AUC = 0.811).	[56]
Tusat (2025)	PNI; HALP score	PNI: albumin + lymphocyte count; HALP: hemoglobin, albumin, lymphocytes, platelets	PNI (*p* = 0.027) and HALP score (*p* = 0.007) were significantly lower in complicated appendicitis, while NLR (*p* = 0.003) and PLR (*p* = 0.008) were higher, indicating worse nutritional-inflammatory status in severe disease.	[57]

AUC—Area under the curve; BMI—Body mass index; CI—Confidence interval; CPA—C-reactive protein-to-prealbumin ratio; CRP—C-reactive protein; HALP—Hemoglobin–albumin–lymphocyte–platelet score; NLR—Neutrophil-to-lymphocyte ratio; NPAR—Neutrophil percentage-to-albumin ratio; OR—Odds ratio; PLR—Platelet-to-lymphocyte ratio; PNI—Prognostic nutritional index.

## Data Availability

No new data were created or analyzed in this study. Data sharing is not applicable to this article.

## Supplementary Materials

The following supporting information can be downloaded at: https://www.mdpi.com/article/10.3390/children13030326/s1, Supplementary Material 1 contains: QA, RoB. Figure S1: Risk-of-bias assessment of non-randomized studies: Traffic-light plot; Figure S2: Risk-of-bias assessment of non-randomized studies: Summary plot.

## Abbreviations

The following abbreviations are used in this manuscript:

**Abbreviation**	**Full Explanation**
ACS NSQIP-P	American College of Surgeons National Surgical Quality Improvement Program–Pediatric
ALB	Albumin
AUC	Area Under the Curve (a performance metric for diagnostic/predictive models)
BMI	Body Mass Index
CAR	C-reactive protein-to-Albumin Ratio
CI	Confidence Interval
CPA	C-reactive protein-to-Prealbumin Ratio
CRP	C-reactive protein (a marker of systemic inflammation)
CS	Cross-sectional Study
CT	Computed Tomography
ECM	Extracellular Matrix
ERAS	Enhanced Recovery After Surgery
HALP	Hemoglobin, Albumin, Lymphocyte, and Platelet score
Hb	Hemoglobin
ICD-9	International Classification of Diseases, Ninth Revision
IL-6	Interleukin-6
MMP	Matrix Metalloproteinase
MUAC	Mid-Upper Arm Circumference
NLR	Neutrophil-to-Lymphocyte Ratio
NPAR	Neutrophil Percentage-to-Albumin Ratio
OR	Odds Ratio
PAS	Pediatric Appendicitis Score
PASAP	Pediatric Appendicitis Severity Assessment
PECOS	Population, Exposure, Comparator, Outcome, and Study design
PLR	Platelet-to-Lymphocyte Ratio
PNI	Prognostic Nutritional Index
POC	Prospective Observational Cohort
PRISMA	Preferred Reporting Items for Systematic Reviews and Meta-Analyses
ROBINS-E	Risk of Bias In Non-randomized Studies—of Exposures
ROC	Retrospective Observational Cohort (also refers to Receiver Operating Characteristic in statistics)
TIMP	Tissue Inhibitor of Metalloproteinases
TLC	Total Leukocyte Count (or Total Lymphocyte Count, depending on context in Table 3)
TNF-α	Tumor Necrosis Factor-alpha
TSFT	Triceps Skinfold Thickness
