# Supplementary material for: Nutritional Status as a Risk Factor for Appendiceal Perforation in Pediatric Acute Appendicitis: Systematic Review
Source: Children (Basel). 2026 Feb 26;13(3):326. doi: 10.3390/children13030326 (PMC13025327; doi:10.3390/children13030326)

children

MDPI

Systematic Review

Nutritional status as a risk factor for appendiceal perforation in pediatric acute appendicitis: Systematic Review

Supplemental Figure S1. Risk of bias assessment of non-randomised studies: Traffic-light plot.

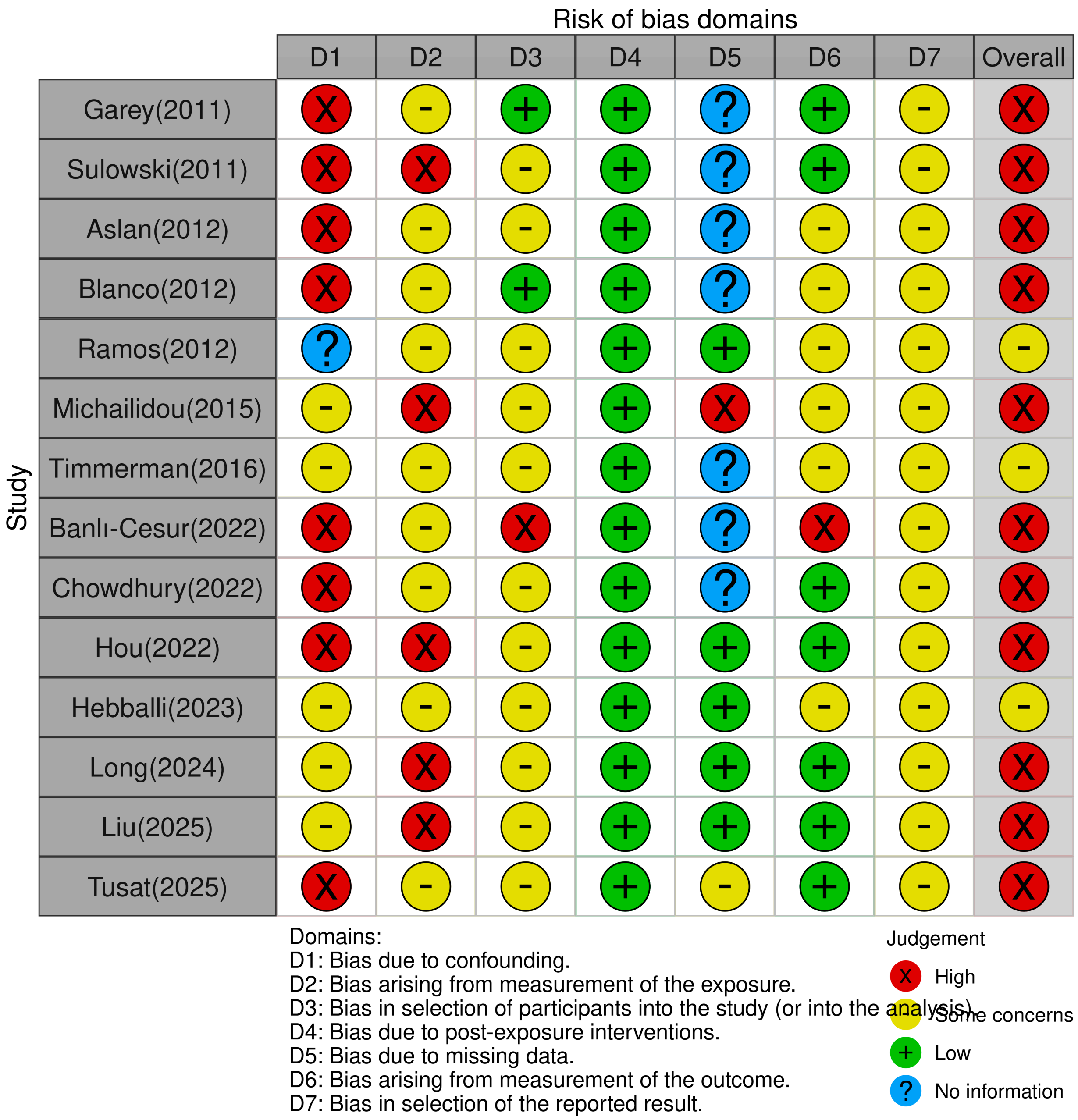

Supplemental Figure S2. Risk of bias assessment of non-randomised studies: Summary plot

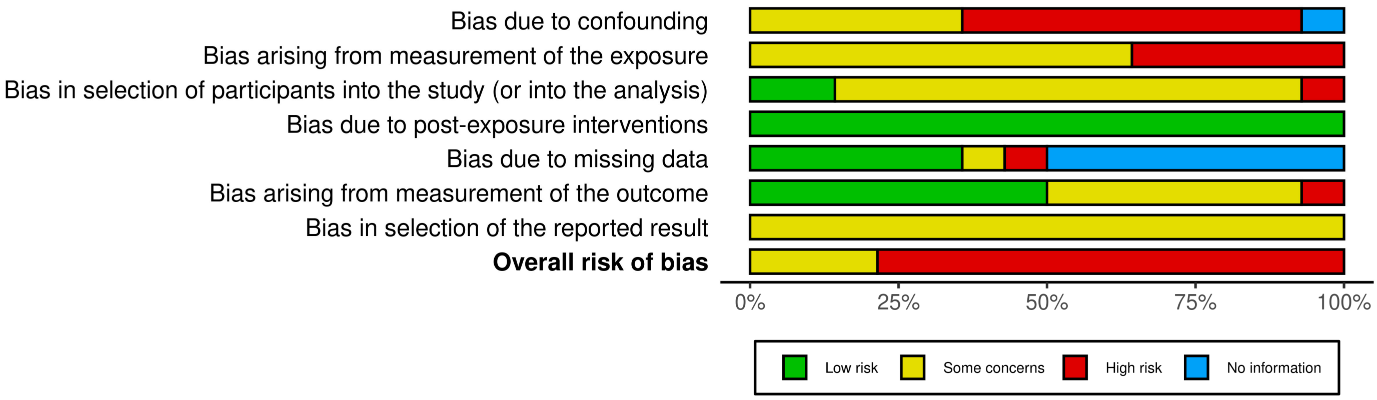

Supplement: Supplementary file 1 [file children-13-00326-s001.zip › children-4165276-supplementary.pdf]
